# Prevalence and Radiographic Morphology of Hallux Valgus in Adolescent Athletes

**DOI:** 10.1002/jfa2.70177

**Published:** 2026-06-16

**Authors:** Yasunari Ikuta, Tomoyuki Nakasa, Minoru Toriyama, Hajime Ito, Ryuya Yamakawa, Mitsuhiro Ochi, Yukio Mikami, Nobuo Adachi

**Affiliations:** ^1^ Department of Orthopaedic Surgery Hiroshima University Hospital Hiroshima Japan; ^2^ Sports Medical Center Hiroshima University Hospital Hiroshima Japan; ^3^ Department of Artificial Joints and Biomaterials Graduate School of Biomedical and Health Sciences, Hiroshima University Hiroshima Japan; ^4^ Department of Rehabilitation Medicine Hiroshima University Hospital Hiroshima Japan

**Keywords:** adolescent athlete, distal metatarsal articular angle, hallux valgus, interphalangeal hallux valgus, morphology, proximal–distal phalanx articular angle

## Abstract

**Introduction:**

Hallux valgus (HV) can shift plantar loading, impair hallux function, and affect athletic performance in adolescent athletes. Although previous studies have focused mainly on dance‐related sports, limited data exist regarding the prevalence and radiographic morphology of HV across broader athletic populations. In particular, the role of hallux interphalangeal alignment and proximal phalanx morphology in deformity severity remains unclear. We aimed to determine HV prevalence among adolescent athletes across various sports and to identify radiographic morphological factors associated with deformity severity.

**Methods:**

This cross‐sectional study included 280 adolescent athletes (188 males and 92 females; mean age, 13.7 ± 1.6 years) across 18 sports. Weight‐bearing foot radiographs were obtained. Radiographic parameters included the HV angle (HVA), intermetatarsal angle (IMA), distal metatarsal articular angle (DMAA), hallux interphalangeal angle (HIA), proximal–distal phalanx articular angle (PDPAA), Meary angle, calcaneal pitch angle, and medial cuneiform–fifth metatarsal height. Multivariable linear regression analyses were performed separately for dominant and nondominant feet to identify factors associated with combined deformity severity (sum of HVA and HIA).

**Results:**

HV was identified in 42.9% of athletes when defined as HVA ≥ 15° in either foot and was more prevalent in female than male athletes (59.8% vs. 34.6%, *p* < 0.001). In contrast, 93.6% of athletes had an HIA ≥ 10° in either foot, based on the conventional adult‐derived threshold, with no significant sex difference. In this multi‐sport adolescent athlete population, distal first metatarsal morphology (DMAA) and proximal phalanx articular morphology (PDPAA) showed the strongest independent associations with combined deformity severity. IMA and female sex were also significant factors, whereas sagittal plane alignment, body mass index, and ankle activity score were not.

**Conclusion:**

This multi‐sport study extends previous findings on adolescent hallux deformity by demonstrating that deformity severity is associated not only with metatarsal structural factors but also with proximal phalanx articular morphology. These findings support a broader radiographic characterization of adolescent HV, although their clinical significance requires further study.

## 1 Introduction

Hallux valgus (HV) is a common forefoot deformity in children and adolescents, with reported prevalence rates of 7%–46% in the general youth population [1, 2, 3]. During adolescence, rapid skeletal growth combined with increased sport‐specific loading may influence foot alignment and contribute to early deformity development [4]. In competitive adolescent athletes, HV can shift plantar loading patterns, impair hallux function, and negatively affect performance, supporting the clinical relevance of early detection [5, 6]. Therefore, understanding the prevalence and structural features of HV is important for characterizing foot morphology in athletes during skeletal maturation.

The literature examining HV in adolescent athletes is limited and has predominantly focused on ballet and dance sport populations [7, 8], reporting a high prevalence of HV, particularly among female athletes; moreover, some studies have documented active deformity progression during adolescence [8, 9]. Nevertheless, the generalizability of these findings remains uncertain because loading patterns and mechanical demands vary substantially across sports. Therefore, evidence from athletes participating in a broader range of sports are needed to determine whether HV is also common beyond dance‐focused athletic populations. Additionally, several prior reports relied on clinical or photographic assessments rather than weight‐bearing radiography, limiting detailed bone morphology evaluation [8, 10].

Previous studies have demonstrated that juvenile HV is anatomically distinct from the deformity typically observed in adults. In fact, adult HV is characterized by progressive metatarsophalangeal (MTP) joint subluxation and an increased intermetatarsal angle (IMA). Conversely, juvenile HV often preserves joint congruency and exhibits morphological variations such as an increased distal metatarsal articular angle (DMAA) [11, 12, 13]. Although these structural features have been described, most prior work has focused on metatarsal alignment, with limited attention to phalangeal morphology. Consequently, the radiographic characteristics of adolescent HV, particularly those involving both metatarsal and phalangeal morphology, are not fully defined. This gap is relevant because combined deformity may involve not only the first metatarsal but also hallux interphalangeal alignment and proximal phalanx articular morphology. Parameters such as the hallux interphalangeal angle (HIA) and proximal–distal phalanx articular angle (PDPAA) have been only minimally investigated, despite evidence that early deformity may involve variations in both the proximal phalanx and first metatarsal [11, 14]. Additionally, radiographic factors associated with deformity severity in adolescent athletes remain poorly understood. Clarifying these factors is essential for characterizing HV during skeletal maturation and identifying structural risk factors relevant to sport participation.

This study aimed to determine the prevalence of HV among adolescent athletes across various sports and to identify radiographic morphological factors associated with HV severity, with particular attention to both metatarsal and phalangeal morphology.

## 2 Materials and Methods

### 2.1 Participants

This cross‐sectional study enrolled adolescent athletes who underwent annual medical checkups at the Sports Medical Center of Hiroshima University Hospital between 2021 and 2025. For athletes evaluated in multiple years, only data from the first visit were included in the analysis to avoid duplicate within‐participant observations.

Two hundred and eighty athletes (188 males and 92 females; mean age, 13.7 ± 1.6 years; age range, 10–18 years) were included. All participants were selected by the Hiroshima City Sports Association for an elite development program. More than 60% of the athletes had competed in national‐level tournaments. The participants represented a wide range of sports, including archery, badminton, basketball, field hockey, gymnastics, handball, ice hockey, judo, kendo, rugby, sailing, skating, soft tennis, swimming, table tennis, tennis, track and field, and wrestling.

Participants' demographic data, including age, sex, and body mass index (BMI), were retrospectively reviewed. Sports activity levels were assessed using the Ankle Activity Score (AAS), which comprises 53 sports, three work‐related activities, and four general daily activities [15]. Scores range from 0 to 10, depending on the type and intensity of physical activity, with 0 indicating the lowest level and 10 the highest. The dominant leg was defined as the leg the participant reported using to kick a ball toward a target.

Written informed consent was obtained from all participants and their legal guardians. The study was approved by the Ethical Committee for Epidemiology of Hiroshima University (approval no. E‐941) and conducted in accordance with the Declaration of Helsinki.

### 2.2 Radiographic Assessment

Weight‐bearing foot radiographs were obtained in dorsoplantar and lateral views using a standardized acquisition protocol. Participants were examined barefoot while standing in a relaxed bipedal stance with weight distributed evenly between both lower limbs. For the dorsoplantar view, each foot was rested flat on the detector, and the X‐ray beam was angled approximately 15° toward the heel and centered over the midfoot. For the lateral view, participants stood with the lateral aspect of each foot adjacent to the detector, and the X‐ray beam was directed horizontally and centered over the midfoot. All radiographs were obtained by experienced radiographers. All images were acquired with a digital radiographic system and analyzed using ShadeQuest/ViewR‐DG (version 1.26; Fujifilm Medical Co. Ltd., Tokyo, Japan).

**FIGURE 1:**
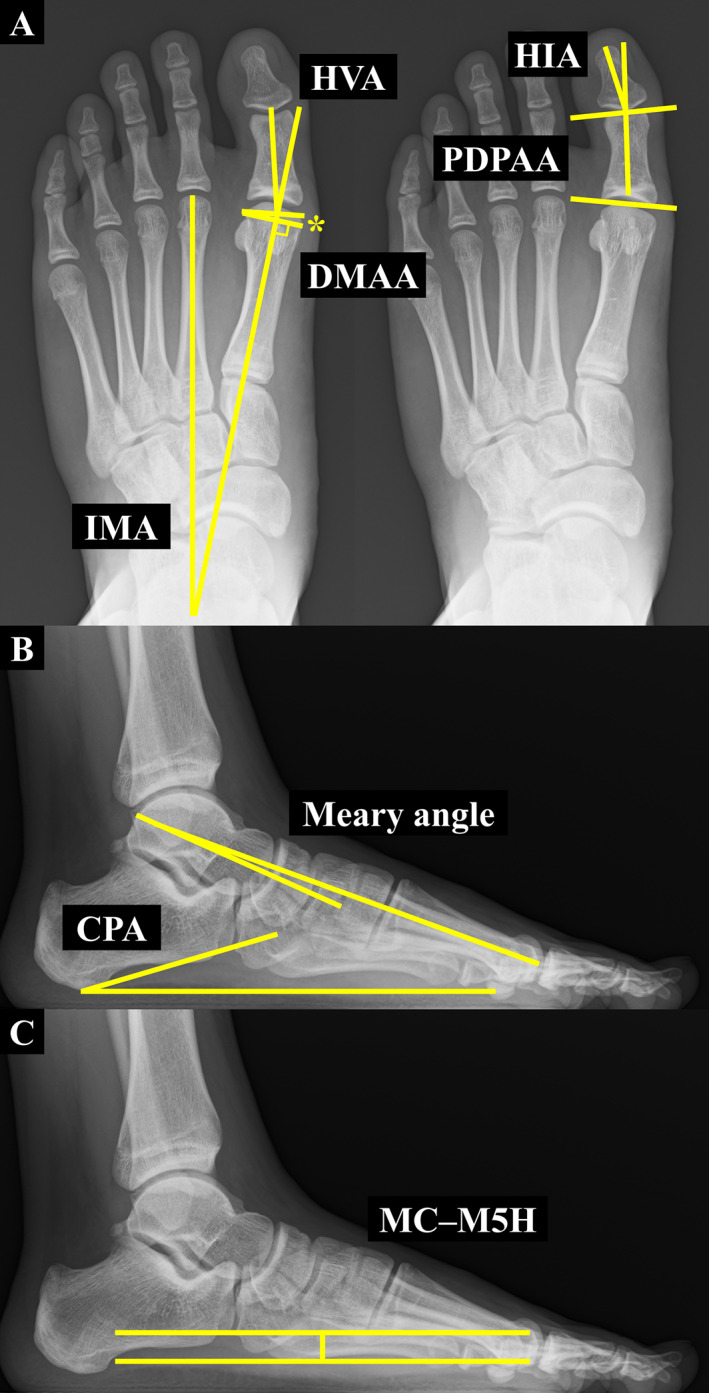
Representative radiographic measurements. (A) Weight‐bearing dorsoplantar radiographs demonstrating measurement of the hallux valgus angle (HVA), intermetatarsal angle (IMA), distal metatarsal articular angle (DMAA), hallux interphalangeal angle (HIA), and proximal–distal phalanx articular angle (PDPAA). PDPAA was defined as the angle between the lines representing the proximal and distal articular surfaces of the proximal phalanx. (B) Weight‐bearing lateral radiograph demonstrating measurement of Meary angle and calcaneal pitch angle (CPA). (C) Medial cuneiform–fifth metatarsal height (MC–M5H).

From the dorsoplantar view, the following parameters were measured: the HV angle (HVA), the angle between the longitudinal axes of the first metatarsal and the proximal phalanx; the IMA, the angle between the longitudinal axes of the first and second metatarsals; the HIA, the angle between the longitudinal axes of the proximal and distal phalanges; the DMAA, the angle between the longitudinal axis of the first metatarsal and the line connecting the distal medial and lateral points of the metatarsal head articular surface; and the PDPAA, the angle between the lines representing the proximal and distal articular surfaces of the hallux proximal phalanx [16]. The lateral talo–first metatarsal angle (Meary angle), calcaneal pitch angle (CPA), and medial cuneiform–fifth metatarsal height (MC–M5H) were assessed. The Meary angle was defined as the angle between the longitudinal axes of the talus and the first metatarsal; the CPA as the angle between the inferior border of the calcaneus and the supporting surface; and the MC–M5H as the vertical distance from the inferior border of the medial cuneiform to the base of the fifth metatarsal. Representative radiographic measurement methods are shown in Figure 1.

HV was defined as HVA ≥ 15° as per established radiographic criteria [17]. For descriptive purposes, an HIA ≥ 10° was used as a conventional adult‐derived radiographic threshold for increased hallux interphalangeal alignment [18]. This threshold has not been validated specifically for adolescents and was therefore not interpreted as a pathological cut‐off in this population. All measurements were obtained by a single experienced examiner blinded to participant information. Measurement procedures followed previously validated methods for radiographic assessment of foot alignment [19, 20, 21, 22, 23].

### 2.3 Statistical Analysis

A single observer (Y.I.) performed all radiographic measurements according to a standardized protocol. To evaluate intra‐rater reliability, the observer repeated measurements on a random subset of 60 feet (10.7%) after a minimum interval of 6 weeks, blinded to the initial results. Intra‐rater reliability was quantified using the intraclass correlation coefficient (ICC) (3,1), derived from a two‐way mixed‐effects model with absolute agreement for single measurements. Participant‐level prevalence was calculated by defining HV as HVA ≥ 15° in either foot. Similarly, the proportion of athletes with an HIA ≥ 10° in either foot was calculated using the conventional adult‐derived threshold. Sex differences were assessed using the chi‐square test for HVA and Fisher's exact test for HIA. For exploratory analysis, sports were grouped according to sports‐specific loading characteristics into jumping/pivoting, combat, and non‐contact/individual sports. Differences across sport‐loading categories were assessed using the chi‐square test. Multivariable linear regression analysis was conducted with the sum of HVA and HIA as the dependent variable. Age, sex, BMI, AAS, and other radiographic parameters were included as independent variables. Because both feet were evaluated in each participant, multivariable regression analyses were performed separately for the dominant and nondominant feet to avoid treating paired limb data from the same participant as independent observations. Statistical significance was set at *p* < 0.05. Multicollinearity was assessed using the variance inflation factor (VIF), with values > 10 indicating problematic multicollinearity. Model fit was evaluated using the adjusted R‐squared value. Model assumptions were checked using residual plots and a normal Q–Q plot. All analyses were performed using IBM SPSS Statistics for Windows version 27.0 (IBM, Armonk, NY).

## 3 Results

**FIGURE 2:**
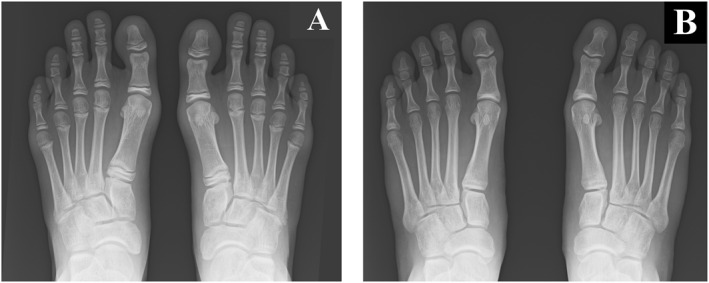
Representative weight‐bearing dorsoplantar radiographs demonstrating skeletal maturity stages. (A) A 13‐year‐old male with open physes at the base of the first metatarsal and proximal phalanx. (B) A 14‐year‐old female with closed physes at the corresponding sites.

The distribution of sports disciplines is summarized in Table 1. The mean BMI and AAS were 20.3 kg/m^2^ and 6.6, respectively. Intra‐rater reliability testing of 60 feet demonstrated good‐to‐excellent reliability across all radiographic parameters (ICC, 0.797–0.941), supporting the use of single‐rater measurements for the primary analyses (Table S1). At the participant‐level, HV was identified in 120 of 280 athletes (42.9%) when defined as HVA ≥ 15° in either foot. Female athletes showed a higher prevalence than male athletes (55/92, 59.8% vs. 65/188, 34.6%; odds ratio, 2.81; 95% CI, 1.68–4.70; *p* < 0.001). In contrast, 262 athletes (93.6%) had an HIA ≥ 10° in either foot, based on the HIA threshold, with no significant sex difference (83/92, 90.2% vs. 179/188, 95.2%; *p* = 0.12; Table 2). In the exploratory sport‐loading category analysis, participant‐level HV prevalence and the proportion exceeding the HIA threshold did not differ significantly across categories (Table 2). Sport‐specific descriptive prevalence data for all 18 sports are provided in Table S2. The mean HVA of the dominant and nondominant feet was 13.4° and 13.0°, respectively, whereas the mean HIA was 13.6° and 13.9°, respectively. Although six of the nine radiographic parameters differed significantly between sides, the absolute differences were small and may be of limited clinical relevance. Descriptive statistics for all radiographic parameters for dominant or nondominant feet are presented in Table 3. HV (HVA ≥ 15°) was identified in 99 (35.4%) and 92 (32.9%) feet on the dominant and nondominant sides, respectively. In contrast, 238 (85.0%) dominant feet and 242 (86.4%) nondominant feet exceeded the HIA threshold (Figure 2). The median HIA was 13.4° in dominant feet and 13.8° in nondominant feet. The distribution of HIA is summarized to provide descriptive data for adolescent athletes, given that adolescent‐specific normative reference values for HIA have not been clearly established (Table S3).

**TABLE 1:** Distribution of sports among participants (*n* = 280).

Sport	*n* (%)
Archery	17 (6.1)
Badminton	4 (1.4)
Basketball	17 (6.1)
Field hockey	24 (8.6)
Gymnastics	11 (3.9)
Handball	10 (3.6)
Ice hockey	9 (3.2)
Judo	11 (3.9)
Kendo	20 (7.1)
Rugby	41 (14.6)
Sailing	14 (5.0)
Skating	15 (5.4)
Soft tennis	13 (4.6)
Swimming	20 (7.1)
Table tennis	19 (6.8)
Tennis	18 (6.4)
Track and field	9 (3.2)
Wrestling	8 (2.9)
Total	280 (100)

**TABLE 2:** Participant‐level prevalence of HV and the proportion of athletes with HIA ≥ 10° by sex and sport‐loading category.

Variable	*n*	HV, *n* (%)	*p* value	HIA ≥ 10°, *n* (%)	*p* value
Overall	280	120 (42.9)		262 (93.6)	
Sex			< 0.001		0.12
Male	188	65 (34.6)		179 (95.2)	
Female	92	55 (59.8)		83 (90.2)	
Sport‐loading category			0.50		0.49
Jumping/pivoting	166	68 (41.0)		157 (94.6)	
Combat	39	20 (51.3)		37 (94.9)	
Non‐contact/individual	75	32 (42.7)		68 (90.7)	

*Note:* Values are presented at the participant‐level. HV was defined as HVA ≥ 15° in either foot. HIA ≥ 10° indicates athletes with an HIA at or above the conventional adult‐derived threshold of 10° in either foot. Sex differences were assessed using the chi‐square test for HV and Fisher's exact test for HIA. Differences across sport‐loading categories were assessed using the chi‐square test. Sport‐loading category analyses were exploratory.

Abbreviations: HIA, hallux interphalangeal angle; HV, hallux valgus.

**TABLE 3:** Comparison of radiographic parameters between the dominant and nondominant feet.

	Dominant foot (*n* = 280)	Nondominant foot (*n* = 280)	Mean difference (dominant—nondominant), 95% CI	*p* value
HVA (°)	13.4 ± 4.9	13.0 ± 4.9	0.41 (0.05 to 0.78)	0.027
IMA (°)	9.2 ± 2.1	9.7 ± 2.1	−0.49 (−0.66 to −0.31)	< 0.001
HIA (°)	13.6 ± 3.7	13.9 ± 3.8	−0.32 (−0.7 to 0.06)	0.095
DMAA (°)	3.5 ± 3.0	3.1 ± 3.1	0.47 (0.18 to 0.76)	0.002
PDPAA (°)	10.7 ± 3.6	11.1 ± 3.7	−0.44 (−0.77 to −0.11)	0.010
Meary angle (°)	2.5 ± 6.8	2.2 ± 6.6	0.31 (−0.25 to 0.86)	0.280
CPA (°)	18.3 ± 4.4	18.7 ± 4.5	−0.38 (−0.68 to −0.09)	0.012
MC–M5H (mm)	10.1 ± 3.8	9.7 ± 3.9	0.42 (0.10 to 0.73)	0.010
HVA + HIA (°)	27.0 ± 6.0	26.9 ± 6.2	0.09 (−0.44 to 0.63)	0.730

*Note:* Values are presented as mean ± SD. Mean differences are shown as dominant–nondominant with 95% confidence intervals. The *p*‐values were calculated using paired *t*‐tests.

Abbreviations: CI, confidence interval; CPA, calcaneal pitch angle; DMAA, distal metatarsal articular angle; HIA, hallux interphalangeal angle; HVA, hallux valgus angle; IMA, intermetatarsal angle; MC–M5H, medial cuneiform–fifth metatarsal height; *n*, number; PDPAA, proximal–distal phalanx articular angle; SD, standard deviation.

For the dominant foot (*n* = 280), the model demonstrated good fit (adjusted *R* ^2^ = 0.393) and no evidence of problematic multicollinearity (all VIFs < 2.35). DMAA, PDPAA, IMA, and sex were significant predictors (all *p* ≤ 0.001), whereas age, BMI, AAS, Meary angle, CPA, and MC–M5H were not significant. Female sex was associated with a higher HVA + HIA sum than male sex (adjusted difference 2.58°, *p* < 0.0001). Residual diagnostics suggested no major violations of model assumptions, with only minor deviations in the tails.

Similarly, in the nondominant foot (*n* = 280), the model demonstrated good fit (adjusted *R* ^2^ = 0.428) and no concerning multicollinearity (all VIFs < 2.40). DMAA, PDPAA, IMA, and sex were significant predictors of the HVA + HIA sum (all *p* < 0.001), whereas age, BMI, AAS, Meary angle, CPA, and MC–M5H were not significant. Female sex was associated with a higher HVA + HIA sum than male sex (adjusted difference 2.24°, *p* = 0.0005). Residual diagnostics indicated no major violations of model assumptions, with only minor deviations in the tails.

Among the radiographic predictors, the DMAA and PDPAA consistently demonstrated the largest standardized coefficients in both the dominant and nondominant feet. Table 4 presents the regression coefficients.

**TABLE 4:** Multivariable linear regression predicting the sum of HVA and HIA.

Predictor	*β* (95% CI)	*p* value	Standardized *β*
Dominant foot (*n* = 280)			
Age (years)	0.0038 (−0.38 to 0.39)	0.99	0.0010
Sex (female vs. male)	2.58 (1.32 to 3.82)	< 0.0001^*^	0.20
BMI (kg/m^2^)	0.057 (−0.10 to 0.21)	0.46	0.038
AAS	0.141 (−0.14 to 0.42)	0.32	0.049
IMA (°)	0.60 (0.29 to 0.90)	0.0002^*^	0.20
DMAA (°)	0.91 (0.71 to 1.10)	< 0.0001^*^	0.46
PDPAA (°)	0.68 (0.52 to 0.85)	< 0.0001^*^	0.41
Meary angle (°)	−0.11 (−0.24 to 0.011)	0.074	−0.13
CPA (°)	0.092 (−0.06 to 0.24)	0.24	0.068
MC–M5H (mm)	0.081 (−0.13 to 0.29)	0.45	0.052
Nondominant foot (*n* = 280)			
Age (years)	0.19 (−0.20 to 0.57)	0.34	0.05
Sex (female vs. male)	2.24 (0.98 to 3.46)	0.0005^*^	0.17
BMI (kg/m^2^)	0.063 (−0.095 to 0.22)	0.43	0.039
AAS	0.18 (−0.096 to 0.46)	0.20	0.062
IMA (°)	0.61 (0.32 to 0.91)	< 0.0001^*^	0.21
DMAA (°)	0.91 (0.72 to 1.11)	< 0.0001^*^	0.46
PDPAA (°)	0.76 (0.60 to 0.91)	< 0.0001^*^	0.46
Meary angle (°)	−0.12 (−0.25 to 0.012)	0.074	−0.12
CPA (°)	0.046 (−0.10 to 0.19)	0.54	0.034
MC–M5H (mm)	0.045 (−0.17 to 0.26)	0.67	0.028

*Note:* Sex was coded as female or male (positive values indicated higher HVA + HIA in females). Standardized *β* indicates the relative strength of association within the model.

Abbreviations: BMI, body mass index; CI, confidence interval; CPA, calcaneal pitch angle; DMAA, distal metatarsal articular angle; HIA, hallux interphalangeal angle; HVA, hallux valgus angle; IMA, intermetatarsal angle; MC–M5H, medial cuneiform–fifth metatarsal height; *n*, number; PDPAA, proximal–distal phalanx articular angle.

^*^ Significant predictors are indicated by (*p* < 0.05).

## 4 Discussion

This study characterized the prevalence and morphological features of HV in adolescent athletes. HV was common across a wide range of sports, with a participant‐level prevalence of 42.9% and a foot‐level prevalence of 32%–36% based on an HVA ≥ 15°; notably, more than 80% of feet exceeded the conventional adult‐derived HIA threshold of 10°. Furthermore, detailed radiographic evaluation identified DMAA, PDPAA, and IMA as independent contributors to deformity severity, indicating that both metatarsal and phalangeal morphology play important roles in adolescent HV. Therefore, early HV may arise from multiple structural components, including metatarsal alignment and phalangeal morphology.

The participant‐level prevalence of HV in this multi‐sport adolescent athlete population was consistent with previous reports in young populations. This extends existing athlete‐based literature, which has largely focused on ballerinas and dance sport competitors, in whom both high HV prevalence and progression during adolescence have been documented [3, 7, 8, 9]. The findings of the present study indicate that HV is not limited to sports involving turnout or extreme forefoot loading. Multivariable regression analysis identified distal first metatarsal and phalangeal morphology as the primary determinants of combined deformity severity (HVA + HIA). Notably, this contrasts with adult HV, which is typically characterized by first metatarsal malalignment; in adolescents, osseous morphology warrants specific evaluation. These results support recent evidence that juvenile HV has distinct morphology, underscoring the need to assess both metatarsal and phalangeal components in adolescents [12, 14, 24, 25, 26].

The exploratory sport‐loading category analysis did not show significant differences in participant‐level HV prevalence among jumping/pivoting, combat, and non‐contact/individual sports. Similarly, the proportion exceeding the HIA threshold was high across all categories. These findings suggest that HV in adolescent athletes may not be explained by simplified sport categories alone. Sport‐specific descriptive data showed variability among individual sports; however, formal sport‐by‐sport comparisons were not performed because several sports included small numbers of participants. Therefore, these findings should be interpreted as exploratory and hypothesis‐generating, and future studies with larger sport‐specific cohorts and detailed exposure data are needed to clarify sport‐specific risk.

In addition to these sport‐related observations, the high proportion of feet with HIA ≥ 10° should be interpreted cautiously, as this threshold is adult‐derived and has not been validated for adolescents; the finding therefore corresponds to a structural distribution rather than pathological deformity. Several alternative interpretations should be considered: an HIA greater than 10° may represent a normal developmental variant in active adolescents, potentially attributable to sport‐related adaptation, growth‐related morphology, or physeal alignment during skeletal maturation. The HIA distribution, including percentile values, is provided as descriptive data to inform future adolescent‐specific reference values and re‐evaluation of the conventional adult‐derived threshold. The sex‐specific findings further support this interpretation: HV defined by HVA ≥ 15° was more prevalent in female athletes, whereas the proportion exceeding the HIA threshold was high in both sexes and did not differ significantly between female and male athletes. This discrepancy suggests that valgus alignment at the MTP joint and hallux interphalangeal alignment may have different demographic or developmental correlates. However, clinically meaningful thresholds should be established only in relation to symptoms, function, and longitudinal progression.

Our observation that PDPAA contributes to deformity severity aligns with recent evidence emphasizing the importance of interphalangeal alignment in juvenile‐onset HV. Chang et al. reported that interphalangeal HV is significantly more common in juvenile‐onset than in adult‐onset HV, with juvenile cases exhibiting greater HIA [14], while studies of adolescent dancers indicate that HV is prevalent and progressive, exacerbated by training load and altered plantar pressure on the vulnerable forefoot [5, 8, 9, 27]. In contrast, studies in adults have emphasized different structural components, such as DMAA and sesamoid position [28], whereas first metatarsal protrusion distance and metatarsus primus adductus angle have been reported as key juvenile HV discriminators [29]. Discrepancies across studies, including inconsistent associations between dance participation and HV [7, 8, 27], may result from methodological heterogeneity in population characteristics, case definitions, and exposure assessment. Together, these findings suggest that sport‐related loading may interact with intrinsic morphology, and that the relative contribution of each factor may vary across populations and methodologies. Although IMA, DMAA, and female sex have been reported previously in juvenile or adolescent HV, the present study extends these findings to a multi‐sport adolescent athlete population and identifies PDPAA as an additional structural correlate of combined deformity severity. This highlights proximal phalanx articular morphology as a potentially overlooked component of adolescent hallux deformity.

These results have several implications for the radiographic assessment of hallux alignment in adolescent athletes. The high proportion of feet exceeding the conventional HIA threshold suggests that assessment focused solely on the MTP joint may underestimate the structural pattern of deformity, and that the interphalangeal joint should also be considered, particularly in young athletes with forefoot symptoms or deformity [7, 14, 24]. When radiographic evaluation is clinically indicated, detailed measurement of DMAA, IMA, PDPAA, and HIA may help characterize deformity severity [11, 12, 24, 26]. Accordingly, clinical considerations such as monitoring during skeletal maturation, footwear modification, load management, and targeted exercise should be regarded as hypotheses for future investigation.

This study has some limitations. First, the cross‐sectional design precluded causal inference. Although we identified associations between morphology and deformity, we could not determine whether specific structural features or sport participation contributed to the development or progression of HV. Second, all participants were drawn from a single elite development program, which may introduce selection bias and limit the generalizability of our findings to recreational athletes or non‐athletic adolescents. Third, the study population consisted exclusively of Japanese adolescent athletes from a single geographic region, and differences in ethnicity, foot morphology, footwear, and training environments may restrict the applicability of these results to other populations. Fourth, symptoms, functional limitations, and participation restrictions were not assessed; therefore, the present findings describe radiographic morphology rather than symptomatic pathology, and clinical relevance cannot be inferred. Finally, detailed sport‐specific exposure data, including duration of sports participation, training volume, and footwear characteristics, were not systematically collected, limiting the assessment of the association between sport‐related exposure and hallux alignment or morphology. In addition, sport‐loading categories were simplified and could not account for event‐specific demands within each sport. Future studies incorporating patient‐reported outcomes, functional measures, detailed exposure data, and longitudinal follow‐up are warranted to determine whether these radiographic features are associated with symptoms, functional impairment, or deformity progression.

## 5 Conclusion

Hallux deformity is common among adolescent athletes across a wide range of sports. A high proportion of feet exceeded the conventional adult‐derived HIA threshold of 10°, although this should be interpreted as a structural distribution rather than a pathological interphalangeal deformity. Radiographic analysis indicated that deformity severity was associated not only with metatarsal structural factors, including DMAA, but also with proximal phalanx articular morphology, represented by PDPAA. These findings suggest that adolescent hallux deformity involves both metatarsal and phalangeal morphology, although the clinical significance of these radiographic features requires further study.

## Author Contributions

**Yasunari Ikuta:** conceptualization, methodology, formal analysis, investigation, data curation, visualization, writing – original draft, writing – review and editing. **Tomoyuki Nakasa:** methodology, writing – review and editing. **Minoru Toriyama:** data curation, writing – review and editing. **Hajime Ito:** data curation, writing – review and editing. **Ryuya Yamakawa:** data curation, writing – review and editing. **Mitsuhiro Ochi:** supervision, writing – review and editing. **Yukio Mikami:** supervision, writing – review and editing. **Nobuo Adachi:** project administration, writing – review and editing.

## Funding

The authors have nothing to report.

## Ethics Statement

The study was approved by the Ethical Committee for Epidemiology of Hiroshima University (Approval No. E‐941).

## Consent

Written informed consent was obtained from all participants and their legal guardians.

## Conflicts of Interest

The authors declare no conflicts of interest.

## Supporting information


**Table S1:** Intra‐rater reliability of radiographic parameters (*n* = 60 feet).

**Table S2:** Sport‐specific descriptive prevalence of HV and the proportion of athletes with HIA ≥ 10°.

**Table S3:** Distribution of hallux interphalangeal angle in adolescent athletes.

## Data Availability

The datasets analyzed during the current study are available from the corresponding author on reasonable request.
