# Supplementary material for: Prevalence and Radiographic Morphology of Hallux Valgus in Adolescent Athletes
Source: J Foot Ankle Res. 2026 Jun 16;19(2):e70177. doi: 10.1002/jfa2.70177 (PMC13272110; doi:10.1002/jfa2.70177)
Supplement: Supplementary file 1 — Table S1: Intra‐rater reliability of radiographic parameters (n = 60 feet). [file JFA2-19-e70177-s003.docx]

**Supplementary Table S1.** Intra-rater reliability of radiographic parameters (n = 60 feet)

| Radiographic parameters | Intraclass correlation | 95% Confidence intervals |
| --- | --- | --- |
| HVA (°) | 0.932 | 0.890–0.959 |
| IMA (°) | 0.902 | 0.840–0.941 |
| HIA (°) | 0.920 | 0.870–0.952 |
| DMAA (°) | 0.797 | 0.681–0.873 |
| PDPAA (°) | 0.913 | 0.858–0.948 |
| Meary angle (°) | 0.828 | 0.728–0.893 |
| CPA (°) | 0.933 | 0.891–0.960 |
| MC–M5H (mm) | 0.941 | 0.903–0.964 |

ICC values were reported as ICC (3,1) with 95% confidence intervals (two-way mixed-effects model, absolute agreement, and single measurements).

Abbreviations: CPA, calcaneal pitch angle; DMAA, distal metatarsal articular angle; HIA, hallux interphalangeal angle; HVA, hallux valgus angle; ICC, intraclass correlation coefficient; IMA, intermetatarsal angle; MC–M5H, medial cuneiform–fifth metatarsal height; n, number; PDPAA, proximal–distal phalanx articular angle.
