# Supplementary material for: Prevalence and Radiographic Morphology of Hallux Valgus in Adolescent Athletes
Source: J Foot Ankle Res. 2026 Jun 16;19(2):e70177. doi: 10.1002/jfa2.70177 (PMC13272110; doi:10.1002/jfa2.70177)
Supplement: Supplementary file 2 — Table S2: Sport‐specific descriptive prevalence of HV and the proportion of athletes with HIA ≥ 10°. [file JFA2-19-e70177-s001.docx]

**Supplementary Table S2. Sport-specific descriptive prevalence of HV and the proportion of athletes with HIA ≥10°**

| **Sport-loading category** | **Sport** | **n** | **HV, n (%)** | **HIA ≥10°, n (%)** |
| --- | --- | --- | --- | --- |
| Jumping/pivoting | Badminton | 4 | 1 (25.0) | 4 (100.0) |
|  | Basketball | 16 | 9 (56.3) | 16 (100.0) |
|  | Field hockey | 24 | 9 (37.5) | 23 (95.8) |
|  | Gymnastics | 11 | 5 (45.5) | 10 (90.9) |
|  | Handball | 10 | 4 (40.0) | 10 (100.0) |
|  | Ice hockey | 10 | 1 (10.0) | 10 (100.0) |
|  | Rugby | 41 | 13 (31.7) | 39 (95.1) |
|  | Soft tennis | 13 | 4 (30.8) | 11 (84.6) |
|  | Table tennis | 19 | 15 (78.9) | 18 (94.7) |
|  | Tennis | 18 | 7 (38.9) | 16 (88.9) |
| Combat | Judo | 11 | 6 (54.5) | 11 (100.0) |
|  | Kendo | 20 | 10 (50.0) | 18 (90.0) |
|  | Wrestling | 8 | 4 (50.0) | 8 (100.0) |
| Non-contact/individual | Archery | 17 | 10 (58.8) | 15 (88.2) |
|  | Sailing | 14 | 5 (35.7) | 14 (100.0) |
|  | Skating | 15 | 7 (46.7) | 14 (93.3) |
|  | Swimming | 20 | 7 (35.0) | 17 (85.0) |
|  | Track and field | 9 | 3 (33.3) | 8 (88.9) |
| **Total** |  | **280** | **120 (42.9)** | **262 (93.6)** |

Values are presented at the participant level. HV was defined as HVA ≥15° in either foot. HIA ≥10° indicates athletes with exceeding the conventional adult-derived HIA threshold of 10° in either foot. Formal sport-by-sport statistical comparisons were not performed because several sports included small numbers of participants.

Abbreviations: HIA, hallux interphalangeal angle; HV, hallux valgus.
