# Supplementary material for: Prevalence and Radiographic Morphology of Hallux Valgus in Adolescent Athletes
Source: J Foot Ankle Res. 2026 Jun 16;19(2):e70177. doi: 10.1002/jfa2.70177 (PMC13272110; doi:10.1002/jfa2.70177)
Supplement: Supplementary file 3 — Table S3: Distribution of hallux interphalangeal angle in adolescent athletes. [file JFA2-19-e70177-s002.docx]

**Supplementary Table S3. Distribution of hallux interphalangeal angle in adolescent athletes**

| **Parameter** | **Dominant feet (n=280)** | **Nondominant feet (n=280)** |
| --- | --- | --- |
| Mean ± SD, ° | 13.7 ± 3.7 | 13.8 ± 3.8 |
| Median [IQR], ° | 13.4 [5.0] | 13.8 [4.9] |
| Range, ° | 5.1–28.0 | 2.6–25.2 |
| 75th percentile, ° | 15.9 | 16.3 |
| 90th percentile, ° | 18.2 | 18.6 |
| 95th percentile, ° | 19.7 | 20.9 |
| Feet with HIA ≥10°, n (%) | 238 (85.0) | 242 (86.4) |

HIA ≥10° was used as a conventional adult-derived radiographic threshold for descriptive purposes and was not interpreted as a pathological cut-off in adolescents.

Abbreviations: SD, standard deviation; IQR, interquartile range; HIA, hallux interphalangeal angle.
